# Clinical Outcome Following Oral Potentially Malignant Disorder Treatment: A 100 Patient Cohort Study

**DOI:** 10.1155/2013/809248

**Published:** 2013-07-09

**Authors:** A. Diajil, C. M. Robinson, P. Sloan, P. J. Thomson

**Affiliations:** ^1^Oral & Maxillofacial Surgery, School of Dental Sciences, Framlington Place, Newcastle upon Tyne NE2 4BW, UK; ^2^Pathology Department, Royal Victoria Infirmary, Newcastle upon Tyne NE1 4LP, UK

## Abstract

Oral potentially malignant disorders (PMDs) are at risk of transforming to invasive squamous cell carcinoma (SCC), but controversy exists over their management and the precise role of interventional treatment. In this study, a cohort of 100 patients presenting with new, single oral dysplastic PMD lesions were followed for up to 10 years following laser excision. PMDs presented primarily as homogeneous leukoplakias on floor of mouth and ventrolateral tongue sites and showed mainly high-grade dysplasia following analysis of excision specimens. Sixty-two patients were disease-free at the time of the most recent followup, whilst 17 experienced same site PMD recurrence, 14 developed further PMDs at new sites, 5 underwent same site malignant transformation, and 2 developed SCC at new oral sites. Whilst laser excision is an effective therapeutic tool in PMD management, prolonged patient followup and active mucosal surveillance together with clear definitions of clinical outcomes are all essential prerequisites for successful interventional management. Multicentre, prospective, and randomised trials of PMD treatment intervention are urgently required to determine optimal management strategies.

## 1. Introduction

Oral potentially malignant disorder (PMD) is the preferred WHO term to describe a number of mucosal lesions which demonstrate an increased risk of squamous cell carcinoma (SCC) development compared with apparently normal oral mucosa. The list of mucosal pathology considered potentially malignant includes discrete lesions such as leukoplakia and erythroplakia, as well as more widespread conditions such as proliferative verrucous leukoplakia, immunodeficiency, oral submucous fibrosis, and perhaps more controversially oral lichenoid lesions [1]. 

Whilst a vast literature exists describing the aetiology, clinical appearance, and the identifiable histopathological features of dysplasia seen in PMD, there remain no universally agreed clinical management protocols. We have described previously, however, both the diagnostic accuracy of obtaining definitive histopathology specimens and the treatment efficacy of the entire lesion removal by interventional laser surgery, and it is now generally accepted that PMD excision is probably the optimal management option [1–3]. 

It remains impossible, unfortunately, to predict either the behaviour of individual PMD lesions or the progress of disease in a particular patient, and some authors raise concerns that formal PMD excision is not proven to prevent SCC development, although it remains a not unreasonable hypothesis [2, 3].

Of perhaps more significance is the lack of clarity regarding overall clinical outcome following PMD treatment and a need to both rationalise terminology and define a more structured patient follow-up regime. The aim of this paper, therefore, is to report on the detailed clinical outcome and followup of a cohort of 100 PMD patients who all underwent a standardised interventional laser surgery treatment to excise dysplastic single lesion disease and whose postoperative progress was documented for up to 10 years following the first presentation.

## 2. Materials and Methods

### 2.1. Patients

Following ethical committee approval and informed patient consent, 100 consecutive PMD patients attending the Maxillofacial Oncology/Dysplasia clinics at Newcastle upon Tyne in Northern England over a 3-year period and who underwent CO_2_ laser excision of dysplastic lesions were recruited to the study. All were new patients, with no prior history of oral cancer or precancer and no previous surgical or radiotherapy treatment, and all presented with distinct, single-site PMD lesions proven on incisional biopsy to exhibit dysplasia. 

Laser surgery was carried out by the same operator (P. J. Thomason) working to a standardised protocol, which has been previously documented, and which comprised formal excision of mucosal lesions and widespread ablation of mucosal margins [2, 3]. The influence of risk factor behaviour such as smoking and alcohol use was identified and appropriate cessation advice was given prior to treatment. All patients were reviewed on a regular basis postlaser intervention, at varying intervals between 1 and 12 months based upon the severity of individual clinical and pathological features, to monitor the clinical course of disease and patients' outcome. The identification of new mucosal disease, biopsy for histopathological diagnosis, and further interventional treatment was carried out in accordance with defined management protocols [2, 3].

All excision biopsy specimens underwent standardised histopathology examination by two experienced oral pathologists (C. M. Robinson and P. Sloan) working to agreed diagnostic criteria. Lesions were graded using both the 2005 World Health Organisation (WHO) classification [4] and a binary grading system (high grade versus low grade) that benefits from increased levels of interobserver agreement and improved predictive value [5]. The two pathologists independently assessed the biopsy material, and discordant grading was resolved by review and consensus. The size of dysplastic lesions was assessed by multiplying the length by width of laser excised specimens as recorded in histopathology reports.

### 2.2. Clinical Outcome

Clinical outcome for each patient was defined at the time of their most recent followup appointment using one of the following terms: *Clinical Resolution*, a patient clinically free of PMD disease following treatment, *Persistent Disease*, whereby the PMD lesion persisted at the same site despite interventional treatment, *Recurrent Disease*, when a PMD lesion recurred at the same site following previously successful excision, *Further Disease*, distinguishing PMD lesion development at new oral sites following previously successful excision, *Malignant Transformation*, whereby invasive SCC arose at the same site of a clinically recognised oral precursor lesion, and *Oral Cancer Development*, in which invasive SCC development occurred but at new oral sites distant from previously recognised or treated precursor lesions.

### 2.3. Statistical Analysis

Statistical analyses were performed using SPSS, version 19.0 (Statistical Package for the Social Sciences, Chicago, IL, USA). Categorical variables for clinical outcome data were summarized and presented descriptively using frequencies and percentages with the chi-square test or Fisher's exact test used to evaluate relationship between variables. Continuous variables were expressed as mean ± standard deviation and were compared using independent Student's *t*-test for pairwise comparison; Kruskal-Wallis test was used for group comparison. Spearman's correlation was used to find and evaluate correlation between variables. The Kaplan-Meier survival analysis method with log-rank test was used to assess the differences between outcome groups and to calculate cumulative disease-free survival rates. For all tests, *P* values ≤0.05 were considered statistically significant.

## 3. Results

Sixty-eight male patients (age range 30–81 years; mean 58 years) and 32 female patients (age range 33–94 years; mean 59 years) comprised the study cohort. Eighty-six patients were either current or ex-smokers, whilst 83 regularly consumed alcohol. One hundred lesions were formally excised by laser, the majority (76) appearing clinically as leukoplakias (67 homogeneous and 9 nonhomogeneous), and the remaining were classified as erythroleukoplakias (16) and erythroplakias (8). Most lesions (79) presented on the floor of mouth and ventrolateral tongue, as summarised in Table 1.

Following consensus WHO grading of the excision specimens, 42 of the lesions were classified as mild dysplasia, whilst the remainder showed moderate dysplasia (26), severe dysplasia (21), or carcinoma in situ (11) (Kappa value = 0.644, *P* < 0.001). Consensus classification using the binary grading system confirmed 56 of the moderate, severe, and carcinoma-in-situ groups as “high grade” and 44 (42 mild and 2 moderate) as “low-grade” lesions (Kappa value = 0.756, *P* < 0.001). 

Following laser excision of their PMD lesions, patients were reviewed for between 2 and 10 years with a mean followup of around 5 years. Nearly two-thirds of cases (62 patients) were completely disease-free following laser surgery, whilst 17 had developed recurrent (same site) disease, 14 further (new site) disease, 5 same site malignant transformation, and 2 developed SCC at new oral sites distinct from their original presenting PMD. No cases in this patient cohort exhibited persistent disease following laser excision. 

A number of clinicopathological features were examined in detail for significance in relation to the documented clinical outcome for the 100 PMD patients.

### 3.1. Age

Chi-square testing revealed no significant relationship between patient age and observed clinical outcome (*P* = 0.361), although middle-age patients (41 to 62 years of age) were predominant in all outcome groups. No SCC development at new sites was seen in patients younger than 40 years.

### 3.2. Sex

Although more male patients presented with PMD lesions, there were no statistically significant associations seen between sex and treatment outcomes in this study (*P* = 0.811; chi-square test).

### 3.3. Clinical Appearance

The vast majority of lesions in this study were leukoplakias, and there were no significant clinical outcome differences discernible between these and lesions with erythroplakic or erythroleukoplakic appearance (*P* = 0.234, Fisher's exact test). Whilst clinical appearance was not significantly related to histopathological diagnosis, non-homogeneous leukoplakia did show higher rates for both recurrent and further dysplastic lesions compared to homogeneous lesions (*P* = 0.016, chi-square test).

### 3.4. PMD Site

Most lesions presented on the floor of the mouth and/or ventro-lateral tongue, with a significant relation seen between clinical outcome and anatomical site of origin (*P* = 0.020, chi-square test), whereby the majority of recurrent and further dysplastic disease cases were seen on the floor of the mouth and ventral tongue. The single dysplastic retromolar lesion in this study underwent malignant transformation, whilst new site SCC development only occurred in patients presenting initially with ventro-lateral tongue lesions.

### 3.5. PMD Lesion Size

A significant relation was found between clinical outcome and PMD lesion size, categorised as <200 mm^2^, between 200 and 600 mm^2^ and >600 mm^2^ (*P* = 0.010, chi-square test). Clinical resolution was most commonly seen in minor and intermediate sized lesions. A higher mean size of presenting lesion was seen in patients developing recurrent disease (393.63 mm^2^) compared to recurrence-free (281.70 mm^2^), albeit nonsignificant (*P* = 0.356, independent *t*-test). Further (new site) dysplastic disease was significantly more common following intermediate sized precursor lesion excision (*P* = 0.049, chi-square test).

Although (same site) malignant transformation was more common following intermediate sized lesion excision, this was not statistically significant (*P* = 0.593, Chi-Square test). However, risk estimate showed that if initial dysplasia size exceeded or equalled 425 mm^2^ (equivalent to the third quartile), the odds ratio for transformation was 2 times higher than that of smaller sized lesions (95% CI, 0.365–11.582).

SCC development distant from primary lesion sites was only seen in intermediate or major sized lesions, and although nonsignificant (*P* = 0.104, Fisher's exact test), there was a definite trend for lesions >200 mm^2^ to exhibit further disease, malignant transformation, and SCC development following treatment.

### 3.6. Smoking Behaviour

The vast majority of patients in this study were either current or ex-smokers, and a significant relation was found between smoking status and clinical outcome (*P* = 0.014, chi-square test), whilst the incidence of both recurrent and further disease was the highest in patients exposed to tobacco, there was a trend for nonsmokers to risk malignant transformation and particularly SCC development, whereby new site carcinomas were seen exclusively in nonsmokers.

Nonsmoking patients also presented with significantly larger lesions (mean size 473.20 mm^2^), compared with both ex-smokers (354.25 mm^2^) and current smokers (241.49 mm^2^), *P* = 0.026, Kruskal-Wallis test. Also, Spearman correlation revealed a significant positive correlation between the degree of histopathological grading and lesion size (*r* = 0.272; *n* = 96; *P* < 0.01), whereby increased PMD size was associated with increased dysplasia severity. 

Chi-Square testing, however, showed no significant relation between clinical outcome and the number of cigarettes smoked per day (*P* = 0.139).

### 3.7. Alcohol Use

There were no statistically significant relationships seen between alcohol intake and outcome (*P* = 0.267, chi-square test), although patients consuming regular alcohol posttreatment risked both recurrent and further disease development, whilst all 3 patients who ceased alcohol consumption remained disease-free.

### 3.8. Histopathological Grading

The WHO system showed a significant relationship with defined outcome categories (*P* = 0.003, Chi-Square test); patients exhibiting malignant transformation or new site SCC development displayed were those seen with either severe dysplasia or carcinoma-in-situ in presenting PMDs. 

**Figure 1:**
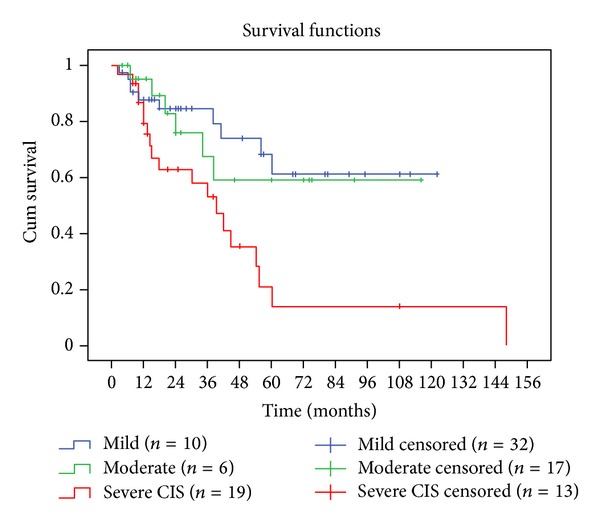
Kaplan-Meier analysis plotting disease-free survival according to the WHO grading of dysplasia (*P* = 0.006, Log-rank test).

Recategorising clinical outcome as either clinical resolution (disease free) or further disease (encompassing recurrent/further PMDs, malignant transformation, or SCC development) emphasised that lesions with severe dysplasia and carcinoma-in-situ were statistically more likely to develop further disease (*P* = 0.010, chi-square test). The degree of dysplasia also had a significant effect on unfavourable outcome, with severe dysplasia/carcinoma-in-situ having a shorter mean time to develop further disease (40 months) compared with moderate (78.8 months) or mild dysplasia (87.83 months). Also, 2- and 5-year disease-free survival rates were much lower for severe dysplasia/carcinoma-in-situ than for either moderate or mild dysplasia (63%, 76%, and 85% and 14%, 59%, and 62%, resp.), *P* = 0.006, Log-Rank test. These data are presented in Figure 1.

**Figure 2:**
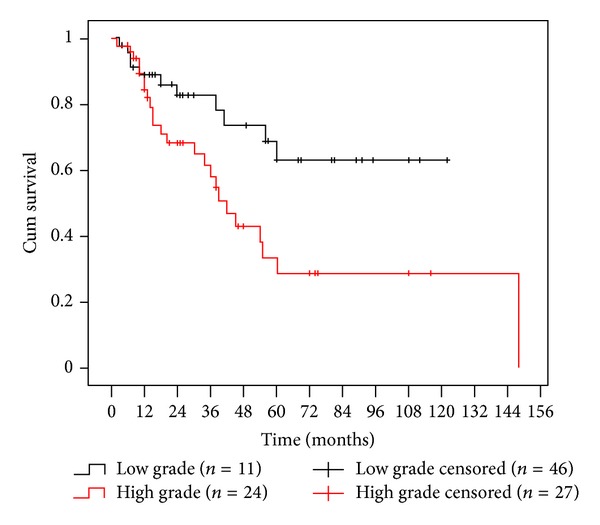
Kaplan-Meier analysis plotting disease-free survival according to high- and low-grade dysplasia (*P* = 0.013, Log-Rank test).

In terms of binary grading, there were demonstrably more high-grade lesions in the outcome groups of recurrent, further disease, malignant transformation, and SCC development, but this was statistically significant only for recurrent disease (*P* = 0.025, Fisher's exact test). Increased statistical significance was seen, however, by recategorising outcome as either clinical resolution or further disease, confirming an increased risk of further disease with high-grade lesions (*P* = 0.021, chi-square test). Patients with high grade dysplasia also had a significantly shorter mean time (64 months) to develop an unfavourable outcome compared to low-grade lesions (88.7 months), and lower 2- and 5-year disease-free survival rates were seen for high-grade compared to low-grade dysplasia (68% versus 83% and 29% versus 63%, resp.), *P* = 0.013, Log-Rank test. These data are summarised in Figure 2.

### 3.9. Laser Excision Margin Analysis

Fourty eight PMD excision specimens had clear margins on histopathological examination with no discernible dysplastic features, whilst in 23 cases foci of mild dysplasia were identified; less commonly, moderate (14) and severe dysplasia (12) or rarely foci of carcinoma-in-situ (3) were reported. Additional intervention for dysplasia positive margin cases was not usually required due to active ablation of all margins at the time of laser excision, although all cases underwent careful clinical surveillance.

Whilst the presence of dysplasia in excision margins did not significantly influence overall postlaser surgery clinical outcome (*P* = 0.053, Fisher's exact test), the majority of patients free from either recurrent (same site) disease or further (new site) disease had clear resection margins, whilst those developing further disease primarily exhibited severe dysplasia in excision margins (*P* = 0.004 and *P* = 0.050, resp., chi-square test).

### 3.10. Length of Followup

**Figure 3:**
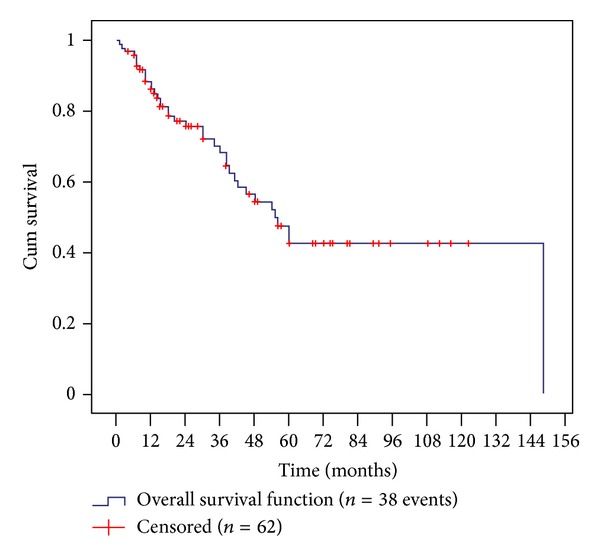
Kaplan-Meier analysis plotting overall disease-free survival.

Clinical outcome in relation to length of followup was determined by plotting PMD-free survival via Kaplan-Meier survival analysis, and this showed a clear relationship with time.  Figure 3 confirms that whilst 88 patients exhibited clinical resolution and were disease-free 1 year after surgery, there was a progressive rise in recurrent and further disease through successive years so that disease-free rates fell to 75 at 2 years, 68 at 3 years, and 47 at 5 years, with only 42 patients PMD-free 10 years after surgery.

**Figure 4:**
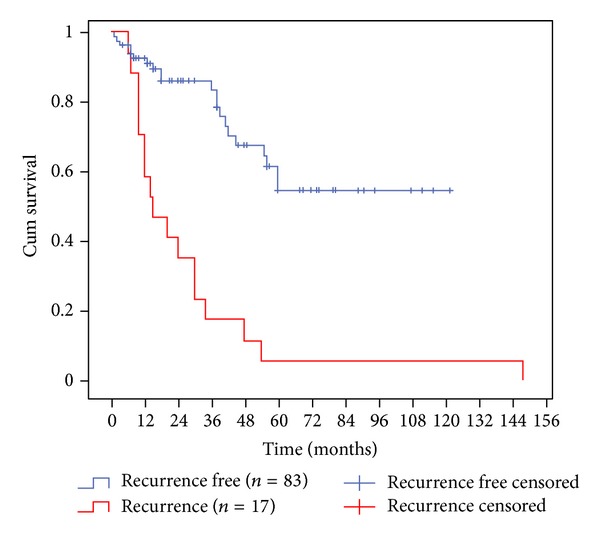
Kaplan-Meier analysis plotting disease-free survival for recurrent (same site) disease and recurrence-free patients (*P* = 0.0001, Log-Rank test).

**Figure 5:**
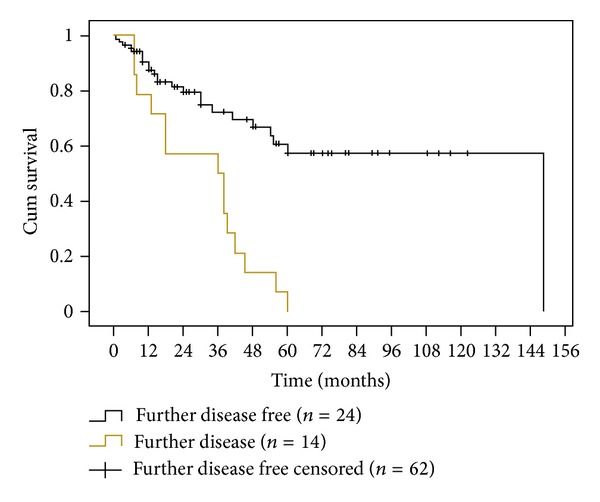
Kaplan-Meier analysis plotting disease-free survival for further (new site) disease and further disease-free patients (*P* = 0.0001, Log-Rank test).

Figure 4 shows that recurrent (same site) PMDs most commonly presented during the first 2 years following laser surgery (11 out of 17 cases), *P* = 0.0001, Log-Rank test. In contradistinction to recurrence, Figure 5 illustrates that further (new site) PMD disease could arise at any time during the first five years of followup, but with particularly significant risk at 1 and 3 years after surgery, *P* = 0.0001, Log-Rank test.

**Figure 6:**
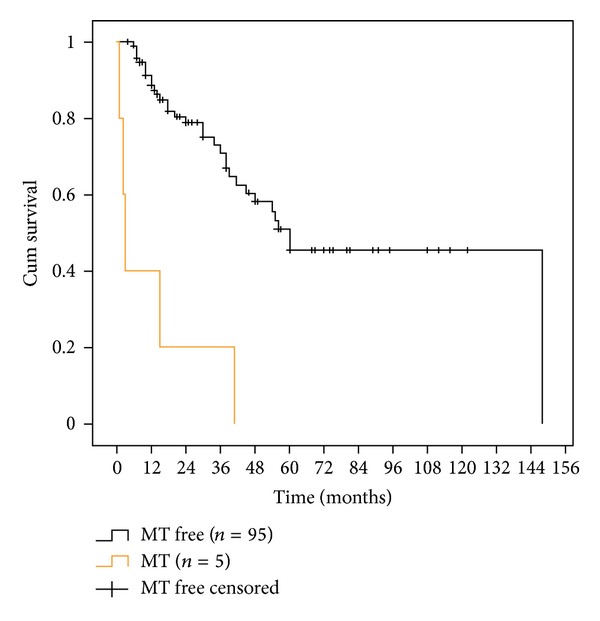
Kaplan-Meier analysis plotting disease-free survival for (same site) malignant transformation and malignant transformation-free patients (*P* = 0.0001, Log-Rank test).

Five malignant transformation cases (same site) occurred during the first 15 months of followup (*P* = 0.0001, Log-Rank test), Figure 6. SCC development (new site cancer) only occurred in 2 cases, both nearly 5 years following severe dysplasia excision from the ventro-lateral tongue. 

### 3.11. Risk Profiling

The clinico-pathological profile of PMD cases observed in each clinical outcome category is summarised in Table 2. Further statistical analysis was performed using univariate and multivariate logistic regression analysis to predict the role of patient age, sex, lesion size, type, histopathology, anatomical site, and resection margin status upon unfavourable clinical outcome (disease active state including recurrent or further dysplasia, malignant transformation, and OSCC development), Table 3.

Whilst patients' age and sex showed no significant effects, non-homogenous leukoplakia was a significant predictor of active disease (*P* = 0.023), increasing risk by nearly 3 times compared to homogenous lesions. Tongue lesions showed a 3.4 increased risk compared with floor of mouth (*P* = 0.013). The presence of severe dysplasia was a highly significant predictor of active disease status (*P* = 0.007); severe dysplasia and carcinoma-in-situ showed a 4.6 and a 4.8 times increased risk, respectively, for active disease compared to mild dysplasia. High-grade dysplasia was also a significant predictor for disease active state (*P* = 0.020), increasing the risk to approximately 3 times that of low-grade dysplasia, Table 3.

The presence of dysplasia in surgical resection margins was also a significant prognostic factor for disease active status (*P* = 0.035), increasing risk by nearly 3 times compared with clear margins. Major sized lesions displayed a 4.5 times increased risk compared to minor sized ones (*P* = 0.045), Table 3. 

## 4. Discussion

The ability to predict clinical outcome for PMDs remains elusive in clinical practice, probably due to lack of understanding of the natural history of the disease, confusion over terminology, limited agreement on therapeutic interventions, and uncertainty regarding patient followup. This paper is unique in presenting detailed, long-term clinical outcome data for a 100 patient cohort presenting with dysplasia-proven PMDs, all excised by laser to a standardised treatment protocol. We also define clinical outcome categories and identify predictive clinico-pathological features. It is notable upon reviewing the literature that many previous authors have not found PMD clinical appearance, anatomical site, histopathological assessment, or features related to patient age, gender, or risk factor behaviour to reliably predict clinical outcome [1, 6–10].

The ultimate goal of PMD diagnosis and management must, of course, is the prevention of SCC. Malignant transformation rates varying widely between 0.1 and 40% have been quoted in the literature, which is extremely unhelpful in individual patient management, although the highest risk of cancer development is believed to occur in the most dysplastic precursor lesions; larger mucosal lesions and nonsmoking patients also appear to be at enhanced risk of malignancy [7, 8, 11, 12].

A number of observational, anecdotal, and retrospective papers have reported clinical outcome and malignant transformation data through the years, but these are significantly weakened by the heterogeneous clinical and histopathological nature of the precursor lesions studied and by a lack of any agreed treatment protocols and uncoordinated follow-up regimes.  Table 4 lists the malignant transformation rates seen in the dysplastic lesions reported in these studies; none of the studies distinguished between same site and new site cancer, but overall malignant transformation rates in excess of 36% (with a mean of 16%) were reported, which are significantly higher than either the 2 new site SCC cases or the 5 same site malignant transformations seen during this study [9, 13–20].

More recently, Mehanna et al. quoted a 12% cancer rate over a mean transformation time of 4.3 years using a systematic review and meta-analysis which included a total of 992 cases although interestingly, because of limitations in the published literature, they only felt able to include 14 papers out of a possible 2837 identified oral precancer publications. The authors reported a lack of high quality evidence to date which limited the scope of their study [21].

To date, there has been a paucity of prospective, randomised controlled trials in oral precancer research, and those that do exist have not fundamentally resolved treatment and clinical outcome dilemmas. This 100 patient cohort study, although not a controlled trial population, is a unique data set facilitating analysis of a defined oral precancer population with shared risk factor behaviour presenting with proven dysplastic PMDs, standardised diagnostic and treatment protocols, consistent clinical decision making, and longitudinal patient observation with documented clinical followup.

Whilst interventional laser excision of mucosal dysplastic lesions appears to reduce the risk of same site malignant transformation, SCC development at new sites remains a risk reflecting field change in PMD disease [2, 3, 10]. Active mucosal surveillance and regular clinic follow up remain mandatory for all PMD patients, and interventional management strategies are best regarded as cyclical, passing from active surgical excision through to surveillance and then returning to surgical intervention for early targeting of further PMD disease. A particular advantage of laser surgery is the ability to repeat excisions or ablations at the same site on a number of occasions, without compromising oral healing or function [2, 10]. 

## 5. Conclusions

In this study, 62% of PMD patients were disease-free following laser excision of dysplastic mucosal lesions, and the risk of malignant transformation remained low at 2 to 5%. The incidence of further disease, however, increased with the length of patient followup, and was higher for non-homogeneous leukoplakias, large mucosal lesions, more severe dysplasias, floor of mouth and ventral tongue sites, and in nonsmoking patients. Long-term patient followup and active clinical surveillance thus remains mandatory for all PMD patients. Multicentre, randomised controlled clinical trials for PMD treatment are now urgently required to determine treatment efficacy.

## Figures and Tables

**Table 1:** Anatomical site of PMD lesions.

Anatomical site	Number of lesions
Floor of mouth	46
Lateral tongue	19
Ventral tongue	14
Soft palate	9
Buccal mucosa	5
Fauces	4
Alveolus	2
Retromolar	1

**Table 2:** Risk profile and clinical outcome.

	Clinical resolution	Recurrent disease	Further disease	Malignant transformation	OSCC development
Number of cases	62	17	14	5	2
Sex					
Male/female	39/23	13/4	10/4	3/2	1/1
Age (Yrs)					
Mean (range)	57 (33–71)	58 (40–77)	59 (39–76)	63 (58–76)	(47–48)
Lesion size (mean mm^2^)	Minor (251)	Major (394)	Major (343)	Intermediate/major (361)	Major (478)
Pathology grading (binary system)					
Low/high	35/27	4/13	6/8	2/3	0/2
Tobacco use^1^	Intermediate	Heavy	Heavy	None	None
Alcohol use^2^	Light	Heavy	Heavy	None	Light

^1^Heavy smoker > 20 cigarettes/day, intermediate smoker 10–20 cigarettes/day, and light smoker < 10 cigarettes/day.

^2^Heavy drinker > 28 units/wk, intermediate drinker 15–28 units/wk, and light drinker < 14 units/wk.

**Table 3:** Logistic regression models for “disease active” status.

Outcome	Risk factors	Univariate analysis		Multivariable analysis	
		Odds (95% CI)	*P* value	Odds (95% CI)	*P* value
*Disease active:* recurrent or further dysplasia, malignant transformation, and OSCC development	Age	1.007 (0.976–1.040)	0.646		
	Sex				
	Females	Reference category			
	Males	1.448 (0.806–3.455)	0.405		
	Leukoplakia types				
	Homogenous	Reference category			
	Nonhomogenous	**2.991 (1.160–7.713)**	**0.023**	3.319 (0.799–13.779)	0.099
	PMDs site				
	FOM	Reference category			
	Tongue	**3.381 (1.292–8.845)**	**0.013**	3.323 (0.775–14.241)	0.106
	Other remaining sites	**2.893 (0.971–8.620)**	**0.057**	0.944 (0.171–5.218)	0.947
	Histopathology (WHO grading)				
	Mid dysphasia	Reference category			
	Moderate	1.129 (0.350–3.641)	0.839	1.960 (0.419–9.167)	0.393
	Severe	**4.622 (1.527–13.990)**	**0.007**	**5.994 (1.282–28.018)**	**0.023**
	CIS	**4.800 (1.123–20.479)**	**0.034**	**17.104 (2.427–120.561)**	**0.004**
	Binary grading				
	Low grade	Reference category			
	High grade	**2.828 (1.182–6.678)**	**0.020**		
	Resection margin				
	Free margins	Reference category			
	Dysplastic margins	**2.812 (1.073–7.371)**	**0.035**	**6.562 (1.545–27.878)**	**0.011**
	PMDs size (mm^2^)				
	Minor < 200	Reference category			
	Intermediate 200–600	2.327 (0.944–5.740)	0.067		
	Major > 600	**4.464 (1.035–18.394) **	**0.045**		

**Table 4:** Malignant transformation of dysplastic precursor lesions.

	Number of dysplastic lesions	Study period (yrs)	Malignant transformation (%)
Silverman et al. (1984) [9]	22	7	36.4
Hogewind et al. (1989) [13]	84	5	3.6
Lumerman et al. (1995) [14]	44	3	16
Schepman et al. (1998) [15]	166	24	12
Cowan et al. (2001) [16]	165	20	14
Holmstrup et al. (2006) [17]	87	20	12
Hsue et al. (2007) [18]	166	10	4.8
Ho et al. (2009) [19]	33	10	24
Arduino et al. (2009) [20]	207	16	7.2
Liu et al. (2011) [12]	138	5	26.8
Warnakulasuriya et al. (2011) [8]	204	9	11.7
Ho et al. (2012) [11]	91	5	25.3
Brouns et al. (2013) [7]	56	4	14.3
